# The effects of unilateral and bilateral cerebellar rTMS on human pharyngeal motor cortical activity and swallowing behavior

**DOI:** 10.1007/s00221-020-05787-x

**Published:** 2020-03-30

**Authors:** Ayodele Sasegbon, Craig J. Smith, Philip Bath, John Rothwell, Shaheen Hamdy

**Affiliations:** 1 Gastrointestinal (GI) Sciences, Division of Diabetes, Endocrinology and Gastroenterology, Faculty of Biology, Medicine and Health, School of Medical Sciences, University of Manchester, Salford Royal Hospital (part of the Manchester Academic Health Sciences Center (MAHSC)), Clinical Sciences Building, Eccles Old Road, Salford, M6 8HD UK; 2 Division of Cardiovascular Sciences, Manchester Centre for Clinical Neurosciences, Lydia Becker Institute of Immunology and Inflammation, University of Manchester, Salford Royal Hospital, Manchester Academic Health Sciences Centre (MAHSC), Salford, UK; 3 Stroke Trials Unit, Division of Clinical Neuroscience, University of Nottingham, Nottingham, UK; 4 Stroke, Nottingham University Hospitals NHS Trust, Nottingham, UK; 5 Sobell Department of Motor Neuroscience and Movement Disorders, University College London, London, UK

**Keywords:** rTMS, Bilateral, Cerebellar, Cerebellum

## Abstract

The cerebellum is recognised to bilaterally modulate sensorimotor function and has recently been shown to play a role in swallowing. Unilateral cerebellar repetitive trans-cranial magnetic stimulation (rTMS) excites corticobulbar motor pathways to the pharynx but the effects of bilateral versus unilateral cerebellar rTMS on these pathways are unknown. In this three-part cross-over study, healthy participants (*n* = 13) were randomly allocated to receive unilateral or bilateral 10 Hz cerebellar rTMS. Participants were intubated with pharyngeal electromyography and/or manometry catheters for motor evoked potentials (MEPs) and pressure recordings. In part 1 of the study, single pulse TMS was used to measure baseline motor cortical pharyngeal MEP (PMEP) and hemispheric cerebellar MEP (CMEP) amplitudes, before cerebellar rTMS was administered. Repeat measures of PMEP amplitude were performed at 15-min intervals for an hour post unilateral and bilateral rTMS. Thereafter, in two further studies, a cortical ‘virtual lesion’ (V/L) was applied prior to cerebellar rTMS with pre and post PMEPs (part 2) and measurements of swallowing accuracy (part 3) using a behavioural task. Compared to baseline, unilateral and bilateral cerebellar rTMS provoked increases in pharyngeal cortical excitation (*P* = 0.028, 0.0005, respectively). Bilateral rTMS was significantly more effective than unilateral in causing cortical excitation (*P* = 0.0005) and in reversing the suppressive neurological (*P* = 0.0005) and behavioural (*P* = 0.0005) effects of a cortical V/L. Our findings suggest bilateral cerebellar rTMS has greater facilitatory effects on corticobulbar motor pathways to the pharynx than unilateral stimulation with the potential to be a more effective clinical therapy if its effects are reproduced in populations with neurogenic dysphagia.

## Introduction

Despite the apparent simplicity of the process of deglutition, each swallow is a physiologically complex activity necessitating fine neurological control, healthy dentition and precisely coordinated contractions of muscles within the head and neck (Sasegbon and Hamdy 2017). Groups of interconnected neurons at multiple locations within the brain stem, cortex and cerebellum fire in synchrony to ensure ingested matter is safely masticated—if needed—, manipulated and propelled through the oral cavity and pharynx on their way towards the stomach (Sasegbon and Hamdy 2017). A normal swallow comprises both conscious and subconscious components. While subconscious control is stereotyped and occurs at a midbrain and cerebellar level, conscious control occurs within sensorimotor areas of the cerebral cortices (Sasegbon and Hamdy 2017).

Functional imaging studies performed during deglutition have shown increased bi-hemispheric activity over cortical motor areas representing swallowing musculature (Harris et al. 2005; Hamdy et al. 1999a, c). Despite bilateral activity, motor swallowing representations show neurophysiologic asymmetry, with one hemisphere having a more active or ‘dominant’ representation than the other in a manner unrelated to an individual’s handedness (Hamdy et al. 1996). The cerebellum—a region of the brain given over to the modulation of (sensori-)motor activity—shares some similarities with the cerebral motor cortices. It too is bi-hemispheric with its own motor homunculus topography (Roostaei et al. 2014). Functional imaging studies have also shown the cerebellum is activated during the process of swallowing (Harris et al. 2005; Hamdy et al. 1999b; Mosier et al. 1999; Suzuki et al. 2003). There has been some suggestion that the cerebellum displays asymmetry during swallowing in a similar manner to the cerebral motor cortex with the left hemisphere of the cerebellum displaying greater functional activity than the right (Malandraki et al. 2009; Suzuki et al. 2003). The functional relevance of this asymmetry is uncertain.

Dysphagia is the term used to characterise swallowing dysfunction. Rather than being a standalone disease, it usually occurs as a consequence of various disease processes (Sasegbon and Hamdy 2017). Strokes are a very common cause of dysphagia, with a yearly incidence of 795,000 in the US alone (Benjamin et al. 2018). Following strokes, up to 65% of patients suffer from post-stroke dysphagia (PSD) (Martino et al. 2005; Benjamin et al. 2018). PSD arises as a result of damage which can occur at any point along the multi-level neuronal pathway needed for swallowing initiation and muscular control (Sasegbon and Hamdy 2017). Imaging studies performed on patients with hemispheric strokes and PSD have shown either reduction or complete loss of activity over cortical motor representations with damage to the ‘dominant’ motor hemisphere—particularly those areas representing pharyngeal musculature—more likely to result in dysphagia (Hamdy et al. 1998). Recovery of swallowing function has been reported to be driven by increased brain activity—indicative of compensatory neuronal plasticity—over the undamaged motor (swallowing) cortical hemisphere, while patients with persistent PSD show little or no evidence of neuronal compensation (Hamdy et al. 1998). PSD has also been shown to occur after infratentorial strokes (Vasant et al. 2019). In this context dysphagia is thought to occur as a result of damage to the midbrain components of the swallowing pathway or disruption of cerebellar-thalamo-cortical neuronal connections.

Repetitive transcranial magnetic stimulation (rTMS) is a neurostimulatory method which has been used to influence the activity of neuronal tissue. Using an electromagnet, magnetic pulses can be delivered at a variety of different frequencies to targeted regions of the brain. In its low frequency form— ≤ 1 Hz (Hz)—it supresses neuronal activity (Mistry et al. 2007), while when delivered at higher frequencies— ≥ 5 Hz—it causes neuronal excitation (Jefferson et al. 2009). Over the past few years, successful attempts at neuromodulation, using techniques such as repetitive transcranial magnetic stimulation (rTMS) have targeted cortical pharyngeal motor areas (Jefferson et al. 2009; Michou et al. 2014). More recently, the realisation of the importance of the cerebellum in the control of swallowing has opened up a new potential therapeutic target for excitatory neurostimulatory interventions.

Jayasekeran et al. (2011) first demonstrated that pharyngeal motor evoked potentials (PMEPs) could be induced following single pulse hemispheric cerebellar TMS delivered to the cerebellar pharyngeal motor areas. Subsequently, Vasant et al. (2015) conducted a study comparing the cortical excitatory effects of different frequencies of cerebellar rTMS delivered to the cerebellar pharyngeal motor areas. It was found that cerebellar rTMS at a frequency of 10 Hz caused the greatest amount of cortical excitation. Curiously, the study did not demonstrate any intrinsic cerebellar excitation, measured as cerebellar MEP amplitude using an intraluminal pharyngeal catheter, as might be expected following administration of an excitatory (10 Hz) cerebellar rTMS intervention.

The cortical virtual lesion protocol developed by Mistry et al. (2007) has been used to test the effectiveness of novel neurostimulatory interventions such as rTMS prior to their use in patients with PSD). They discovered that by applying low frequency (1 Hz) cortical rTMS to the ‘dominant’ pharyngeal cortical representation, neuronal activity could be suppressed for up to an hour post cessation of stimulus (Mistry et al. 2007). Subsequently, Verin et al. (2012). performed a videofluoroscopic study which found a cortical ‘virtual lesion’ produced similar deleterious changes to swallowing as a hemispheric stroke Sasegbon et al. (2019) conducted a virtual lesion reversal study using cerebellar rTMS. The study compared the ability of right or left sided cerebellar rTMS to reverse the effects of a cortical virtual lesion. It was found that 10 Hz cerebellar rTMS was fully able to reverse the suppressive brain and behavioural effects of a cortical virtual lesion. Furthermore, there was no difference in excitatory effect due to whether the cerebellar hemisphere stimulated was unilateral or contralateral to the location of the virtual lesion (Sasegbon et al. 2019).

At present, all studies performed using cerebellar rTMS in the oropharyngeal system have utilised a unilateral hemispheric approach. No study has attempted to compare the brain and behavioural effects of unilateral and bilateral cerebellar rTMS. We, therefore, wanted to examine the effects of these two interventions on the human swallowing motor system with the hypothesis that bilateral hemispheric cerebellar rTMS will have a greater cortical excitatory and behavioural effect than unilateral hemispheric cerebellar rTMS.

## Method

The study was structured as a two-armed multi-protocolled cross over study. The two study arms were unilateral cerebellar rTMS and bilateral cerebellar rTMS. As the primary purpose of the study was to compare and contrast the neurophysiologic and swallowing behaviour effects of bilateral rTMS to unilateral rTMS, no sham rTMS arm was performed. Unilateral rTMS served as an ‘active’ comparator arm.

**Fig. 1:**
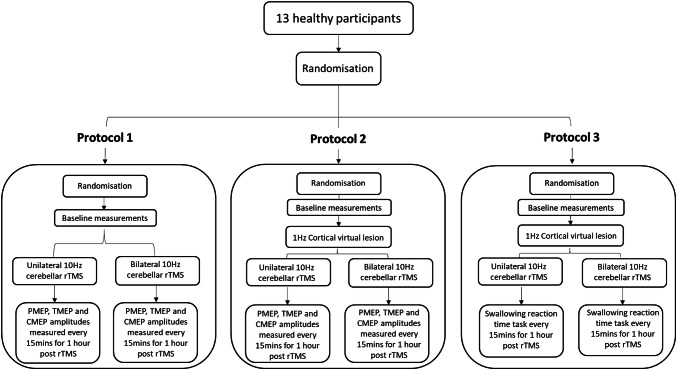
Flow diagram illustrating study protocol

The three study protocols were: (1) Assessing pharyngeal area motor activity, measured as PMEP amplitude; (2) Assessing pharyngeal area motor activity following a cortical ‘virtual lesion’ and (3) Assessing swallowing accuracy following a cortical ‘virtual lesion’. Each healthy participant would across protocols attend the gastrointestinal laboratory on up to six occasions with at least 48 h between attendances (Fig. 1). Participants were randomised prior to the commencement of each protocol. Specifically, the study was designed to first assess the effects of the two interventions in an unperturbed pharyngeal motor system (protocol 1) and then assess effects following a virtual lesion (protocols 2 and 3) examining both the neurophysiologic and behavioural effects of the two interventions.

Ethical approval for this study was granted by the North West NHS research ethics committee (19/NW/0119). All studies were performed in the gastrointestinal laboratories at Salford Royal Hospital NHS Foundation Trust in accordance with the declaration of Helsinki.

### Participant recruitment

Utilising data from previously published rTMS studies in the field of swallowing neurophysiology (Sasegbon et al. 2019; Jefferson et al. 2009; Vasant et al. 2015) (Jayasekeran et al. 2011) it was determined that a minimum of 12 healthy participants would be needed per treatment arm in the PMEP amplitude studies (unilateral and bilateral) to obtain a statistical power of 80% (*P* = 0.05) with an effect size of 40%. Regarding the behavioural study, analysis of previous data (Sasegbon et al. 2019) indicated at least 10 participants per treatment arm were needed to obtain a similar statistical power.

In total, twenty-two healthy adults were recruited with each study protocol containing 13 participants. All participants gave informed written consent after being given a minimum of 24 h to read and consider the information in the participant information sheet. Nine females and four males took part in the two PMEP studies with a mean age of 22 ± 2 years and 23 ± 4 years. Eight females and five males participated in the swallowing behavioural study with a mean age of 23 ± 5 years. Inclusion criteria were broad, with participants being eligible for recruitment as long as they were: not medically unwell; not dysphagic or had ever had previous dysphagia or had any implanted medical devices or metal.

The two primary outcome measures were PMEP amplitude and swallowing accuracy. Changes in cortical PMEP amplitude measured using single pulse TMS have been shown to be correlated with changes in neuronal activity by previous studies in the field (Bohning et al. 1999). Cortical PMEP amplitudes were measured in microvolts (µV) by administering 10 individual pulses of TMS over both hemispheric pharyngeal cortical representations (dominant and non-dominant). Cerebellar PMEP amplitudes were also measured by delivering five TMS pulses over both hemispheric cerebellar pharyngeal representations.

Swallowing accuracy—as measured using a reaction time task—has been previously shown to be correlated with swallowing behaviour (Verin and Leroi 2009; Jayasekeran et al. 2010). It was measured by assessing the number of swallows on target out of a total of 10 attempts during a challenging timed swallowing task.

Secondary outcome measures were MEP latencies. Latencies were measured in milliseconds (ms) from the point of each TMS pulse being delivered to the onset of a MEP.

### Electromyography

Pharyngeal electromyography (EMG) was performed by inserting a thin (3.2 mm in diameter) intraluminal catheter (Gaeltec Ltd, Isle of Skye, UK) containing two platinum bipolar ring shaped electrodes trans-nasally or trans-orally—depending on participant preference—into the pharynx. Catheters were positioned 13–15 cm from the lips or the nostrils and secured in place using medical grade dermal tape. This was in keeping with anatomical pharyngeal EMG catheter positioning described by previous studies (Vasant et al. 2014; Michou et al. 2012; Jayasekeran et al. 2010; Jefferson et al. 2009). An earth skin electrode (H69P, Tyco Healthcare, Gosport, UK) was then placed on each participant’s sternocleidomastoid.

Thenar EMG was performed by placing two skin electrodes (H69P, Tyco Healthcare, Gosport, UK) two centimetres apart on the abductor pollicis brevis of the hand contralateral to each participant’s dominant pharyngeal cortical hemisphere. An earth electrode was placed on their radial prominence. Thenar MEPs (TMEPs) were measured as a control.

All wiring was first connected to a pre amplifier (CED 1902). Subsequently, so as to ensure the MEP signals were free from electrical ‘noise’ (particularly at 50 Hz) pre-amplified signals were passed through two noise cancelling devices (HumBug, Quest Scientific, North Vancouver, Canada). Signals were then sent to a data acquisition interface (Cambridge Electronic Design (CED) micro 1401, UK) before finally being sent to a personal computer (Dell, Gosport, UK). Signals were analysed using Signal Soft software (v4.0, CED, Cambridge, UK).

### Swallowing reaction time task

Swallowing accuracy was determined using a manometry catheter of 1.5 mm diameter (Gaeltec Ltd, Isle of Skye, UK). This was positioned so that its sensor resided at the same pharyngeal location as the EMG electrodes. The catheter was attached to a ‘swallow timer’, a custom built device used and validated in several published studies (Michou et al. 2012; Jefferson et al. 2009; Sasegbon et al. 2019). It times and measures pharyngeal pressures which are then analysed by a custom built swallowing timing program (Medical Physics, SRFT) on the laboratory computer (Dell, Gosport, UK).

### Single pulse transcranial magnetic stimulation

Cortical and cerebellar single pulse TMS was performed using a Magstim 200 stimulator (The Magstim Company, Whitland, UK). It generated a magnetic field strength of 2.2T and was connected to a figure of eight coil 7 cm in diameter. TMS over the cortex was administered by holding the coil over the top of the head pressed flat against the scalp tilted at an angle of 45° from the sagittal plane. Cerebellar TMS was delivered by pressing the coil over the posterior aspect of the skull with its handle pointed superiorly. Cortical and cerebellar PMEP amplitudes were measured by delivering TMS pulses at 120% of resting motor threshold (RMT). As per the design of the figure of eight electromagnetic TMS coils, pulses of magnetic energy are focussed at the intersection between the two halves of the coil.

### Repetitive transcranial magnetic stimulation

Cortical and cerebellar rTMS was administered using a Magstim super‐rapid stimulator with an output of 1.8T (The Magstim Company, Whitland, UK) attached to a figure of eight coil of 7 cm diameter. Coil positioning and orientation was identical to that for single pulse TMS. Low frequency rTMS (as per the virtual lesion protocol (Mistry et al. 2007)) was administered over the dominant cortical pharyngeal representation, 600 pulses at a frequency of 1 Hz and an intensity of 120% pharyngeal RMT. High frequency rTMS was administered over the cerebellum, 250 pulses at a frequency of 10 Hz and an intensity of 80% of pharyngeal RMT capped at 90% of thenar area RMT. Unilateral rTMS was administered over the right hemispheric cerebellar pharyngeal motor representation. Bilateral cerebellar rTMS was administered by serially delivering rTMS over the right cerebellar pharyngeal representation and then the left cerebellar pharyngeal representation.

### Study protocols

#### Protocol 1

Each participant was randomised and asked to attend the gastrointestinal laboratory on two occasions at least 48 h apart. On arriving participants were asked to sit in a padded chair and make themselves comfortable. Subsequently two skin electrodes were placed over the APB of their right hand. A third skin electrode was then placed over their right radial eminence and connected to an earth wire. An intraluminal pharyngeal catheter was then inserted either trans-nasally or trans-orally according to participant preference and positioned as described above (Magara et al. 2016; Vasant et al. 2016; Jefferson et al. 2009). A skin electrode was then placed over the right or left sternocleidomastoid and connected to an earth wire.

To enable identification of bi-hemispheric cortical pharyngeal motor areas, thenar motor areas and cerebellar pharyngeal motor areas, a disposable surgical cap was placed over each participants head and anchored securely. Anatomical landmarks—cranial inion and vertex—were identified and marked on the surgical cap. The cranial vertex was defined as the distance half way along a line measured from the cranial inion to the nasion. Single pulse TMS was then used to identify and mark single locations (Jefferson et al. 2009) corresponding to the: right and left hemispheric cortical pharyngeal motor areas, right and left hemispheric thenar motor areas and right and left hemispheric cerebellar hemispheric pharyngeal motor areas. Each of the aforementioned cortical and cerebellar motor areas was marked as the precise location which when stimulated consistently produced MEPs of the greatest amplitude. Cortical and cerebellar pharyngeal RMTs were defined as the minimum intensity of TMS stimulation required to evoke PMEPs ≥ 20 μV in amplitude in 5 out of a total of 10 trials. Cortical thenar area RMTs were defined as the minimum intensity of TMS required to evoke TMEPs ≥ 50 μV in 5 out of 10 pulses. In keeping with what has been observed regarding cortical swallowing motor asymmetry (Hamdy et al. 1996) the more active or ‘dominant’ pharyngeal motor cortical representation was defined as the representation which required the lowest intensity of TMS to evoke PMEPs of the required amplitude, i.e., the hemisphere with the lower RMT. PMEP amplitudes were measured bilaterally over both cortical and cerebellar hemispheres. TMEP amplitudes were measured only over the cortical hemisphere corresponding to the ‘dominant’ pharyngeal representation.

Baseline measurements of PMEP and TMEP amplitudes were then obtained by delivering 10 TMS pulses over the marked cortical and cerebellar motor areas at an intensity of 120% of each area’s RMT. During each study, participants were asked to try to avoid speaking, coughing, swallowing or clearing their throats. If any of these activities happened during a measurement, the measurement was discarded and repeated.

Participants were then randomly allocated to unilateral (right sided) or bilateral cerebellar rTMS delivered over the cerebellar pharyngeal representations. Random allocation was done using SPSS. Ten Hz cerebellar rTMS was then delivered as per each participant’s allocated group. The 250 pulses were delivered as five blocks of 50 pulses with an inter train wait of 10 s between stimulus blocks as per standard safety guidelines for repetitive brain stimulation (Vasant et al. 2015). Subsequently, repeat cortical and cerebellar amplitude measurements were made using single pulse TMS at 0, 15, 30, 45 and 60 min post rTMS.

#### Protocol 2

Participant randomisation, pharyngeal catheter placement, cap placement, TMS mapping, motor area thresholding, baseline and follow up TMS measurements and cerebellar rTMS were all performed as per protocol 1. However, in protocol 2, a cortical ‘virtual lesion’ was administered after the baseline PMEP measurements to suppress the cortical swallowing system prior to high frequency cerebellar rTMS. This was followed immediately by the cerebellar rTMS [either unilateral (contralesionally to the virtual lesion) or bilateral]. As per Mistry et al. (2007) the virtual lesion was administered by delivering 600 pulses of cortical rTMS at a frequency of 1 Hz and an intensity of 120% RMT over the ‘dominant’ pharyngeal cortical representation. Thereafter the follow-up PMEP measurements were acquired.

#### Protocol 3

As per protocols 1 and 2, participants were randomised, surgical caps were placed on participants’ heads, pharyngeal EMG intraluminal catheters were inserted and single pulse TMS was performed to map cortical and cerebellar motor representations and obtain RMTs.

Swallowing timing was performed as has been described in published studies by Jefferson et al. (2009) and Sasegbon et al. (2019). Participants swallowed ~ 3 ml of water per cued swallow, facilitated by bolus delivery through an orally placed plastic tube connected to a hand held syringe. After motor mapping, the pharyngeal EMG catheter was removed and a manometry catheter inserted and connected to the swallowing timing system (Medical physics, Salford Royal NHS Trust, UK). Calibration was then performed so that the system was able to distinguish between a swallow and the absence of a swallow. During calibration the threshold for swallow registration was set at ≈ 45% of maximal swallowing pressure. Subsequently, the swallow timing software (Medical physics, Salford Royal NHS Trust, UK) was loaded. Participants were first asked to complete 10 normal swallows when prompted by the swallowing program. They were then directed to complete 10 swallows delivered as quickly as they were able. Cues were delivered, audibly via a click emitted by the swallowing timing system at the start of each measurement and visually via a graphic on the computer monitor. A swallow was cued every 10 s. The normal and fast swallow latencies were then used to calculate a challenging graphical target on the computer screen. Participants were then prompted to swallow 10 times and hit the target. The number of swallows on target out of 10—a primary outcome measure—was then recorded.

After baseline swallowing accuracy was recorded, a cortical virtual lesion was administered as per protocol 2. This was followed by either unilateral or bilateral high frequency cerebellar rTMS as per protocols 1 and 2. Following the virtual lesion and cerebellar rTMS repeat swallow performance measurements were made at 0, 15, 30, 45 and 60 min.

### Data analysis

#### Protocol 1–2

Amplitudes—measured as the size of the highest to the lowest MEP peak—and latencies—measured as the time to onset of a MEP—of each group of 10 cortical PMEP traces and each group of five cerebellar PMEP traces were averaged and converted to percentage changes from baseline. Cortical and cerebellar MEP traces were analysed using SPSS Statistics v. 22.0 (IBM Corp., Armonk, NY, USA).

As per previous studies in the field (Vasant et al. 2015; Jefferson et al. 2009), both interventions (unilateral and bilateral rTMS) were compared to each other and also to baseline using repeated‐measures analysis of variance (rmANOVA). For the purposes of rmANOVA the data inputted for each intervention were percentage changes from individual baseline for all participants at each measured timepoint of the study. Post hoc analysis using Bonferroni’s correction was performed.

#### Protocol 3

Swallowing accuracy was measured as number of swallows on target out of ten. All data were then converted to percentage changes from baseline. SPSS was again used as per protocols 1 and 2.

For all three protocols, data are expressed as mean ± standard error of the mean unless stated otherwise. A *P* value of < 0.05 was taken to indicate statistical significance.

## Results

No adverse events arose during the course of the study with cortical or cerebellar single pulse TMS or rTMS.

### Cortical and cerebellar pharyngeal motor area locations

**Fig. 2:**
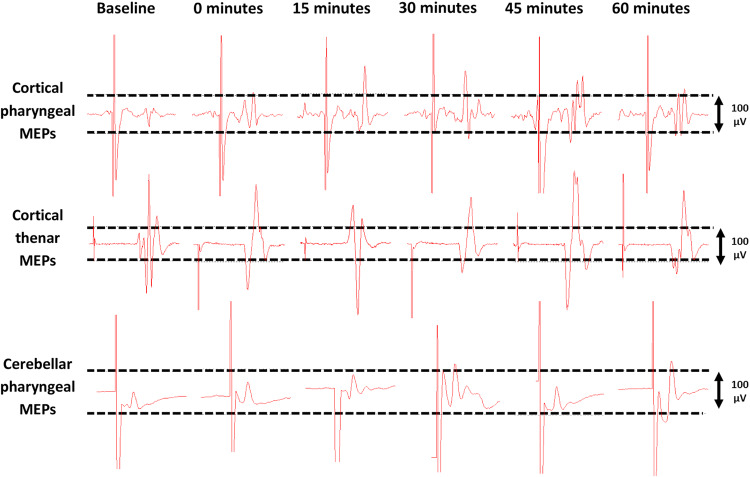
Cortical PMEP and TMEP and cerebellar PMEP traces measured from the dominant pharyngeal cortical hemisphere and the right cerebellar hemisphere for a study participant pre and post unilateral cerebellar rTMS

Single pulse TMS evoked reproducible PMEPs and TMEPs from cortical and cerebellar motor representations (Fig. 2). RMTs for both ‘dominant’ and ‘non-dominant’ cortical pharyngeal areas, cortical thenar motor areas and cerebellar pharyngeal motor areas for protocols 1, 2 and 3 are shown in Table 1.

**Table 1:** Cortical and cerebellar resting motor thresholds (RMT) for protocols 1, 2 and 3

	Unilateral cerebellar rTMS	Bilateral cerebellar rTMS
Protocol 1		
Stronger cortical hemisphere	75 ± 9.0	75 ± 3.0
Weaker cortical hemisphere	85 ± 7.5	85 ± 5.5
Thenar	45 ± 6.5	50 ± 6.0
Cerebellar	55 ± 5.0	55 ± 2.5
Protocol 2		
Stronger cortical hemisphere	75 ± 7.5	75 ± 3.5
Weaker cortical hemisphere	85 ± 7.5	86 ± 5.5
Thenar	44 ± 7.5	53 ± 6.8
Cerebellar	55 ± 5.0	55 ± 2.5
Protocol 3		
Stronger cortical hemisphere	75 ± 7.5	75 ± 8.5
Weaker cortical hemisphere	85 ± 5.0	85 ± 5.0
Thenar	50 ± 9.0	44 ± 10.0
Cerebellar	55 ± 5.0	55 ± 3.0

Across studies, 9/13 participants had more active (‘dominant’) right hemisphere pharyngeal responses, while 4/13 had more active left hemisphere responses. None of the participants studied was observed to change their more active pharyngeal motor hemisphere over the course of the study.

Over the left cortical hemisphere, the mean pharyngeal motor location was (mean ± standard deviation) 4.9 ± 0.81 cm anterior, and 1.50 ± 0.79 cm lateral. Over the right hemisphere, the mean motor location was 4.8 ± 1.1 cm anterior and 2.1 ± 0.72 cm lateral.

Over the left cerebellar hemisphere, the mean pharyngeal motor location was − 3.6 cm inferior (*y* coordinate),  ± 1.4 cm and − 3.1 cm lateral (*x* coordinate),  ± cm. Over the right hemisphere, the mean motor location was − 3.7 cm inferior,  ± 1.2 cm and + 2.6 cm lateral,  ± 1.3 cm.

### Protocol 1: the effects of unilateral and bilateral cerebellar rTMS on cortical activity in an unperturbed system

#### Cortical PMEPs

**Fig. 3:**
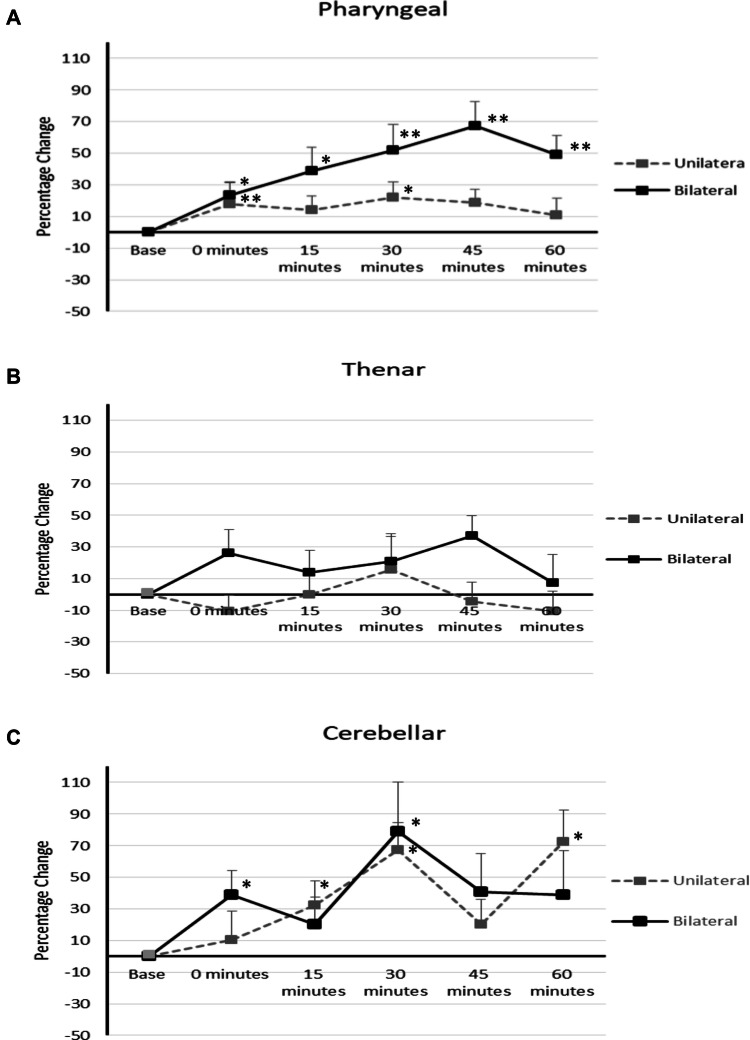
Graphs of PMEP amplitudes showing percentage changes from baseline with unilateral and bilateral cerebellar rTMS. **a** pharyngeal cortical area **b** thenar cortical area **c** cerebellar cortex. Asterisks indicate statistical difference between interventions (*P < 0.05, **P < 0.005). Error bars indicate standard error of the mean

Following both unilateral and bilateral cerebellar rTMS there was no observed difference in the pattern of interhemispheric cortical excitation. As a result, PMEP data from both hemispheres were combined for further analysis. Baseline amplitudes of the PMEPs and TMEPs are shown in Table 2, with % changes in Figs. 2 and 3.

**Table 2:** Baseline cortical pharyngeal, cortical thenar and cerebellar pharyngeal MEP amplitudes in microvolts (µV) for protocols 1 and 2

	Unilateral rTMS	Bilateral rTMS
	MEP amplitudes µV	MEP amplitudes µV
Protocol 1		
Cortical pharyngeal	159.8 ± 154.5	146.7 ± 157.9
Cortical thenar	2183.6 ± 1946.5	1756.4 ± 662.7
Cerebellar pharyngeal	213.6 ± 268.6	190.5 ± 176.3
Protocol 2		
Cortical pharyngeal	163.5 ± 198.4	209.9 ± 166.1
Cortical thenar	1696.0 ± 1179.2	2227.8 ± 1481.2
Cerebellar pharyngeal	155.6 ± 153.9	448.7 ± 288.2

Repeated measures ANOVA revealed a significant Time × Intervention interaction for unilateral and bilateral cerebellar rTMS (*F*_8.711,213.420_ = 5.242, *P* = 0.0005). Main effects of intervention and time were *F*_2,49_ = 17.205, *P* = 0.0005 and *F*_5,45_ = 8.656, *P* = 0.0005. Post hoc analysis showed that bilateral cerebellar rTMS induced greater excitation than unilateral cerebellar rTMS *P* = 0.02 (Fig. 3a). Bilateral rTMS was significantly different to unilateral rTMS at 45 and 60 min, *F*_2,2_ = 22.343, 11.265, *P* = 0.0005, 0.003.

Post hoc analysis of interventions compared to baseline showed both unilateral cerebellar rTMS and bilateral cerebellar rTMS provoked significant increases in pharyngeal area cortical PMEP amplitudes *P* = 0.028 and 0.0005, respectively. One-way ANOVA identified that bilateral cerebellar rTMS was greater than baseline at 0, 15, 30, 45 and 60 min post rTMS, *F*_2,2,2,2,2_ = 4.240, 7.409, 12.00, 22.343, 14.265, *P* = 0.035, 0.001, 0.0005, 0.0005, 0.0005. Unilateral cerebellar rTMS was greater than baseline at 0 and 30 min, *F*_2,2_ = 4.240, 12.00, *P* = 0.049, 0.043.

Cortical PMEPs did not show any significant Time x Interventional differences in post rTMS latencies (*F*_7.534,131.840_ = 1.20, *P* = 0.305) (Table 3).

**Table 3:** Cortical pharyngeal and thenar and cerebellar pharyngeal MEP latencies in milliseconds (ms) for protocols 1 and 2

	Unilateral cerebellar rTMS	Bilateral cerebellar rTMS
Protocol 1		
Cortical pharyngeal (combined) ms		
Baseline	9.1 ± 0.3	9.2 ± 0.4
0 min	9.0 ± 0.7	9.6 ± 0.8
15 min	9.4 ± 0.5	9.0 ± 0.6
30 min	9.2 ± 0.8	9.1 ± 0.6
45 min	8.8 ± 0.6	9.3 ± 0.9
60 min	9.0 ± 0.4	9.1 ± 0.9
Cortical thenar (combined) ms		
Baseline	20.5 ± 1.2	21.7 ± 0.6
0 min	20.3 ± 0.8	21.4 ± 0.6
15 min	21.7 ± 1.0	21.2 ± 0.7
30 min	21.5 ± 0.8	21.4 ± 1.1
45 min	21.6 ± 1.0	21.3 ± 0.3
60 min	22.0 ± 1.2	21.0 ± 0.7
Cerebellar pharyngeal (combined) ms		
Baseline	5.8 ± 0.4	5.5 ± 0.6
0 min	5.7 ± 0.5	5.8 ± 0.3
15 min	5.5 ± 0.6	5.9 ± 1.0
30 min	6.3 ± 0.8	5.1 ± 0.7
45 min	5.8 ± 0.7	5.4 ± 0.7
60 min	5.6 ± 0.6	5.9 ± 0.9
Protocol 2		
Cortical pharyngeal (combined) ms		
Baseline	8.4 ± 0.7	9.5 ± 0.2
0 min	8.6 ± 0.3	9.2 ± 0.6
15 min	8.7 ± 0.5	8.8 ± 0.9
30 min	8.7 ± 0.5	8.8 ± 0.4
45 min	8.7 ± 0.7	9.1 ± 0.7
60 min	8.3 ± 0.4	8.7 ± 0.5
Cortical thenar (combined) ms		
Baseline	21.6 ± 0.4	21.0 ± 0.5
0 min	21.6 ± 1.0	21.1 ± 0.9
15 min	21.5 ± 0.6	21.4 ± 1.2
30 min	21.1 ± 0.6	21.1 ± 0.8
45 min	21.1 ± 0.7	21.0 ± 0.7
60 min	21.7 ± 1.2	20.6 ± 0.7
Cerebellar pharyngeal (combined) ms		
Baseline	7.1 ± 1.7	8.1 ± 0.4
0 min	6.7 ± 0.5	7.7 ± 0.5
15 min	6.9 ± 1.1	7.6 ± 1.2
30 min	6.8 ± 1.1	7.7 ± 0.7
45 min	7.2 ± 0.8	7.6 ± 1.1
60 min	7.0 ± 0.8	7.7 ± 1.1

### Cortical TMEPs

No significant thenar Time × Intervention interaction was found for unilateral and bilateral cerebellar rTMS (*F*_10,160_ = 0.670, *P* = 0.751) (Fig. 3b).

There were no significant Time × Intervention interactions for TMEP latency measurements post bilateral and unilateral cerebellar interventions (*F*_3.034,51.576_ = 1.011, *P* = 0.396) (Table 3).

#### Cerebellar PMEPs

Repeated measures ANOVA showed a significant Time × Intervention interaction for both interventions (*F*_8.025,200.613_ = 5.784, *P* = 0.0005). There were no significant differences in cerebellar PMEP amplitude between unilateral and bilateral cerebellar rTMS *P* = 0.269 (Fig. 3c).

Post hoc analysis comparing each intervention against baseline demonstrated that unilateral and bilateral cerebellar rTMS were able to increase post interventional cerebellar PMEP amplitudes *P* = 0.0005, 0.047. Unilateral rTMS was significantly different to baseline at 15, 30 and 60 min (*F*_2,2,2_ = 3.523, 10.106, 7.443, *P* = 0.041, 0.004 and 0.001). Bilateral cerebellar rTMS was significantly different to baseline at 0 and 30 min (*F*_2,2_ = 4.044, 10.106, *P* = 0.019, 0.001).

No significant differences in latencies were observed for the two interventions (*F*_6.719,117.584_ = 1.621, *P* = 0.104) (Table 3).

### Protocol 2: the comparative effectiveness of unilateral and bilateral cerebellar rTMS at reversing the suppressive effects of a cortical ‘virtual lesion’

#### Cortical PMEPs

As was the case in protocol 1, both hemispheres PMEP data were combined before being converted into percentage changes from baseline.

**Fig. 4:**
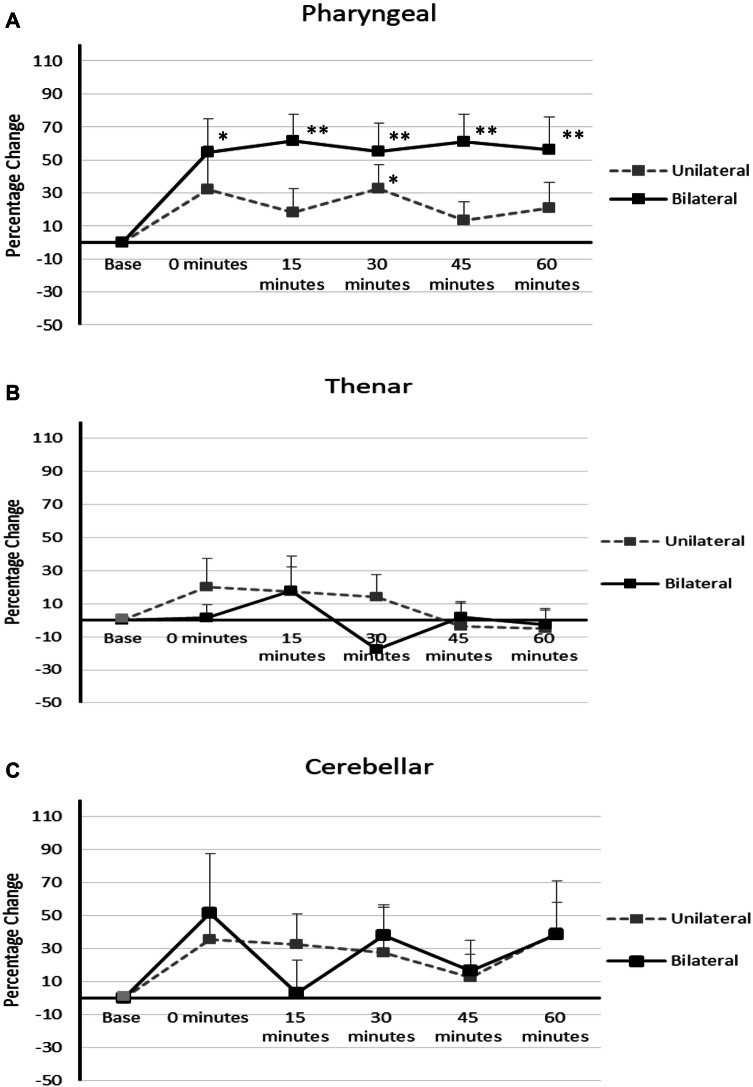
Graphs of PMEP amplitudes showing percentage changes from baseline with unilateral and bilateral cerebellar rTMS following a cortical ‘virtual lesion’. **a** pharyngeal cortical area **b** thenar cortical area **c** cerebellar cortex. Asterisks indicate statistical differences between interventions (*P < 0.05, **P < 0.005). Error bars indicate standard error of the mean

Repeated measures ANOVA revealed significant Time × Intervention interaction for unilateral and bilateral cerebellar rTMS (*F*_6.560,167.285_ = 4.196, *P* = 0.0005). Main effects of rTMS intervention and time were *F*_2,49_ = 12.329, *P* = 0.0005 and *F*_5,47_ = 7.794, *P* = 0.0005. Bilateral rTMS was found to cause a significantly greater increase in PMEP amplitudes compared to unilateral rTMS *P* = 0.025 (Fig. 4a). Furthermore, bilateral cerebellar rTMS was significantly different to unilateral cerebellar rTMS at 15 and 45 min, *F*_2,2_ = 12.386, 11.031, *P* = 0.013 and 0.005.

Post cortical virtual lesion, unilateral and bilateral cerebellar rTMS provoked significant increases in pharyngeal area cortical PMEP amplitude compared to baseline *P* = 0.031, 0.0005. Unilateral cerebellar rTMS was significantly different to baseline at 30 min post rTMS, *F*_2_ = 9.740, *P* = 0.048, while bilateral rTMS was revealed to be different to baseline at 0, 15, 30, 45 and 60 min, *F*_2,2,2,2,2_ = 4.487, 12.386, 9.740, 11.031, 7.627, *P* = 0.018, 0.0005, 0.0005, 0.0005 and 0.001.

Cortical PMEPs did not show any significant differences in post interventional latencies (*F*_8.085,145.534_ = 1.886, *P* = 0.066) (Table 3).

#### Cortical TMEPs

No significant Time × Intervention differences in TMEP amplitudes were seen (*F*_5.979,98.651_ = 1.286, *P* = 0.242) (Fig. 4b).

There were no significant differences in latencies post bilateral and unilateral cerebellar rTMS (*F*_6.578,118.412_ = 0.844, *P* = 0.547) (Table 3).

#### Cerebellar PMEPs

Unlike protocol 1, rmANOVA did not reveal a significant Time × Intervention interaction for rTMS interventions, *F*_6.132,107.303_ = 0.99, *P* = 0.431 (Fig. 4c).

Cerebellar PMEPs did not show any significant Time x Interventional differences in post rTMS latencies (*F*_6.765,121.763_ = 0.419, *P* = 0.884) (Table 3).

### Protocol 3: the effectiveness of unilateral and bilateral cerebellar rTMS at reversing the behavioural effects of a cortical ‘virtual lesion’

**Fig. 5:**
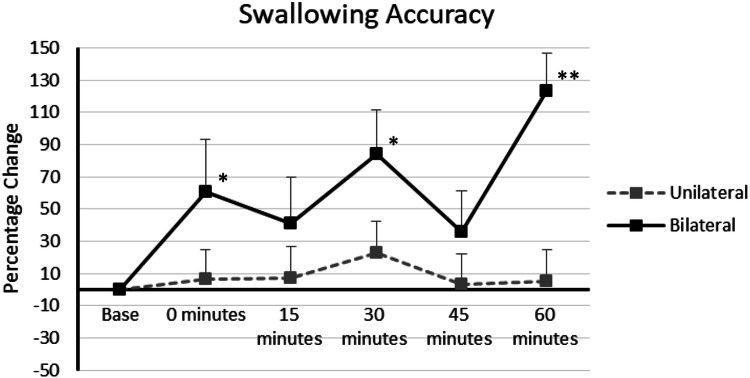
Graph of swallowing accuracy showing percentage changes from baseline with unilateral and bilateral cerebellar rTMS following a cortical ‘virtual lesion’. Asterisks indicate statistical difference between interventions (*P < 0.05, **P < 0.005). Error bars indicate standard error of the mean

At baseline, swallowing accuracy (out of 10) was 3 ± 1 for unilateral and 2 ± 1 for bilateral cerebellar rTMS. After delivery of a cortical ‘virtual lesion’, rmANOVA showed a significant Time × Intervention interaction for unilateral and bilateral cerebellar rTMS, *F*_6.501,172.285_ = 4.720, *P* = 0.0005. Analysis of main effects of intervention and time were *F*_2,49_ = 12.272, *P* = 0.0005 and *F*_5,45_ = 5.863, *P* = 0.0005. Bilateral cerebellar rTMS was observed to result in a significant increase of swallowing accuracy over unilateral rTMS *P* = 0.001 (Fig. 5). Significant differences between bilateral rTMS and unilateral rTMS were seen at 0 and 60 min *F*_2,2_ = 6.326, 19.158, *P* = 0.034, 0.0005.

Comparisons between the interventions and baseline revealed bilateral rTMS caused a significant increase in accuracy *P* = 0.0005. One-way ANOVA showed time specific differences at 0, 30 and 60 min (*F*_2,2,2_ = 6.326, 7.624, 19.158, *P* = 0.003, 0.001 and 0.0005). Despite the visual appearance of increased swallowing accuracy, unilateral rTMS was not significantly different from baseline *P* = 1.00.

## Discussion

Our results show that unilateral and bilateral cerebellar rTMS are able to cause cortical and cerebellar pharyngeal area excitation and reverse the suppressive PMEP and behavioural effects of a cortical ‘virtual lesion’. However, bilateral cerebellar rTMS was found to be more effective in inducing both motor excitation and reversing the effects of a cortical ‘virtual lesion’ than unilateral rTMS.

### Potential mechanisms of cerebellar rTMS neuromodulation

Compared to what is known about cortical swallowing pathways, less is known about the cerebellum and its connections to swallowing centres within the brainstem and cortex. Both functional imaging and neurophysiologic studies suggest the existence of ipsilateral and contralateral connections between each cerebellar hemisphere and cortical motor areas. Indeed, Vasant et al. (2015) performed a dose response cerebellar rTMS study and reported that cerebellar rTMS induced bilateral cortical excitation regardless of the cerebellar hemisphere targeted. Moreover, Sasegbon et al. (2019) showed cerebellar rTMS was able to reverse the inhibited PMEP and behavioural effects of a cortical virtual lesion. In keeping with the previous findings of cerebellar bi-hemispheric cortical connectivity, reversal was observed irrespective of whether rTMS was applied ipsilaterally or contralaterally to the location of the cortical virtual lesion (Sasegbon et al. 2019). Our study provides further evidence in support of these findings. Unilateral and bilateral cerebellar rTMS both resulted in bilateral cortical hemispheric excitation. This was the case both with and without the application of a cortical virtual lesion. These findings can be explained by an understanding of cerebellar efferent neuronal pathways.

The cerebellum is attached to the brainstem by three peduncles through which it communicates with various motor nuclei in the brainstem and motor areas in the cortex (Mottolese et al. 2013; Daskalakis et al. 2004). Contralaterally, the effects of cerebellar targeted rTMS can potentially be explained by rTMS activation of the cerebellar cortex causing subsequent stimulation of dentate nuclei within each cerebellar hemisphere. Efferent axons which arise from the dentate nuclei exit the cerebellum before progressing to the contralateral motor cortex after first synapsing with the thalamus (Daskalakis et al. 2004). Ipsilaterally, a pathway which potentially explains the effects of cerebellar rTMS involves the cerebellar fastigial nuclei. Efferent outflow from the fastigial nuclei interfaces with components of the central pattern generator (CPG) within the brainstem (Krebs 2006; Daskalakis et al. 2004). The CPG is partly responsible for the control of swallowing and interfaces with cortical swallowing representations bilaterally (Jean 2001). Alternately, interhemispheric communication may explain the ipsilateral cortical effects of cerebellar rTMS. Because the cerebellum is an organ which modulates (sensori)motor activity, its effects are predominantly suppressive (Roostaei et al. 2014). Therefore, it is possible rTMS over the cerebellar cortices may cause a decrease in inhibitory outflow and a concurrent increase in cortical activity.

### Effects of unilateral and bilateral cerebellar rTMS on neuro-electrical activity

Our study shows both unilateral and bilateral cerebellar rTMS are able to cause pharyngeal cortical excitation when applied to an unconditioned swallowing motor system. Interestingly, and in keeping with our hypothesis, bilateral cerebellar rTMS was significantly more able to provoke cortical excitation than unilateral rTMS. This greater excitatory effect may be as a result of greater stimulation input into the cerebellum and a subsequent increased effect on cortical pathways. Despite the fact no analogous studies have previously been performed in this field, our study findings are similar to the 2015 findings of Vasant et al. (2015), whereupon unilateral 10 Hz hemispheric cerebellar rTMS was found to provoke pharyngeal cortical excitation when administered without any preconditioning.

Despite the potential confounding of comparing amplitude changes across studies due to the different populations studied and the known variability of rTMS responses, it is interesting to note that the maximal mean cortical excitatory effects in the Vasant study (≈ 60%) (Vasant et al. 2015) were greater than the excitatory effects in the unilateral group of our study (maximally 22%) while being less than the effects of bilateral cerebellar rTMS (maximally 67%).

When viewed together, our study and the earlier study by Vasant et al. (2015) illustrate a path towards optimisation of cerebellar rTMS. Vasant et al. identified that 250 pulses delivered at 10 Hz rather than 1, 5 or 20 Hz was the frequency of cerebellar rTMS most able to cause the greatest degree of excitation; by contrast our study has now shown that a bilateral approach can provoke still greater excitation (Vasant et al. 2015). It should be noted that Vasant et al. (2015) did not show any significant increase in cortical excitability with unilateral 20 Hz cerebellar rTMS. This implies an excitatory frequency threshold. However, in light of the increased excitation produced by bilateral compared to unilateral 10 Hz cerebellar rTMS it may be the case that bilateral cerebellar rTMS at other frequencies could be more excitatory than unilateral rTMS.

Thenar MEPs in both unilateral and bilateral groups did not show any statistically significant evidence of increased excitation in keeping with the findings of previously published studies (Sasegbon et al. 2019; Jefferson et al. 2009; Vasant et al. 2015) which suggests that cerebellar and cortical rTMS can be focally administered without concurrent interaction with other motor areas. This finding suggests that despite the increased total energy being administered to the cerebellum, as a technique, bilateral cerebellar rTMS does not result in any greater degree of stimulus spread.

Cortical pharyngeal and thenar latencies were also in keeping with previously published studies (Sasegbon et al. 2019; Vasant et al. 2015).

### Direct cerebellar effects of rTMS

In protocol 1, cerebellar rTMS was found to cause measurable increases in cerebellar evoked PMEPs implying some increase in intrinsic cerebellar excitability. This was the case for both unilateral and bilateral interventions. This finding is different to that which has been previously described in the literature. Earlier studies by Vasant et al. (2015) and Sasegbon et al. (2019) did not show significant increases in cerebellar PMEPs over baseline. However, both studies did show the appearance (visually) of increased cerebellar excitation. Interestingly, in protocol 1, there was no statistical difference between the effects of unilateral and bilateral rTMS. Though more work is needed to validate and further investigate this observation, it may imply an intrinsic cerebellar energy dependant threshold (for excitation) which is not normally evident when eliciting cortical PMEPs. Curiously, in protocol 2, unilateral and bilateral rTMS were not shown to have a significant Time x Intervention interaction despite the visual appearance of an increase in excitability over baseline. RTMS response variability may explain this observation. Alternately, the suppressive effect of the preceding cortical lesion in protocol 2 may have affected cerebellar PMEP amplitudes. More work is needed to investigate this phenomenon.

### Effects of unilateral and bilateral cerebellar rTMS reversing the PMEP effects of a cortical virtual lesion

Unilateral and bilateral cerebellar rTMS were able to reverse the expected suppressive PMEP effects of a cortical ‘virtual lesion’. Just as in the previous protocol, the excitatory effects of bilateral cerebellar rTMS were greater than unilateral. This protocol did not contain a sham arm which would have served to illustrate the effects of an unopposed ‘virtual lesion’, because its primary aim was to compare the effectiveness of these two techniques against one another. However, because a virtual lesion was administered prior to cerebellar rTMS, the absence of any significant suppression is at least supportive of the reversal of its effect. Furthermore, in keeping with the findings of a previous cerebellar rTMS reversal study (Sasegbon et al. 2019), cerebellar rTMS caused excitation above baseline in a manner akin to published non-virtual lesion studies of cerebellar and cortical rTMS excitation (Vasant et al. 2015; Gow et al. 2004).

With the aforementioned caveats, the maximal mean cortical excitatory effects of cerebellar rTMS post virtual lesion in the Sasegbon study (≈ 30%) (Sasegbon et al. 2019) was similar to our unilateral rTMS post ‘virtual lesion’ study arm (30%) yet less than the effects of bilateral cerebellar rTMS (60%). We can surmise, therefore, that bilateral cerebellar rTMS may be a more potent methodology to restore function, where there is deficit, such as in stroke and other forms of neurologic disorder.

### Effects of unilateral and bilateral cerebellar rTMS reversing the behavioural effects of a cortical virtual lesion

Once more, bilateral cerebellar rTMS was more effective than unilateral stimulation at enhancing the swallowing behavioural effects following a cortical ‘virtual lesion’. When viewed in conjunction with the results of protocols 1 and 2, it can be seen that bilateral cerebellar rTMS has a greater neuro-electrical and behavioural magnitude of effect than unilateral rTMS. This is important, because published studies have showed a correlation between the ability of neuromodulatory interventions to reverse swallowing behavioural changes and observable clinical improvements in PSD patient studies (Jayasekeran et al. 2010). Evidence in support of cerebellar rTMS being a clinically useful tool for dysphagia is limited. However, a recent single patient case report published by Vasant et al. (2019) showed that unilateral cerebellar rTMS applied to a participant with PSD was associated with observable improvements in post interventional PMEP amplitudes and videofluoroscopic measurements of aspiration. Furthermore, continued improvements in swallowing ability were observed in the weeks to months following the intervention. Although more and larger studies need to be done, these findings point towards unilateral cerebellar rTMS having a role in PSD management. Notwithstanding, our study findings now suggest that bilateral rTMS may be a more effective clinical intervention than unilateral cerebellar rTMS in PSD.

Some consideration needs to be given to the role of metaplasticity in the interactions between the effects of the cortical ‘virtual lesion’ and 10 Hz rTMS applied to the cerebellar motor areas. Studies in other motor systems have shown rTMS induced neuronal responses can be modulated by initial priming of a targeted motor region with rTMS (Abraham 2008). However, it is difficult to say whether metaplasticity played a part in our study findings. The amount of excitation observed in protocol 2, where cerebellar rTMS was administered following a ‘virtual lesion’ was comparable to protocol 1, where 10 Hz cerebellar rTMS was delivered in isolation. More work will need to be done in the future to examine metaplasticity in the swallowing motor system.

### Clinical application

RTMS is a neuromodulatory technique with inherent risks. Cortical rTMS carries with it a reported risk of provoking seizure activity (Rossi et al. 2009, Wassermann 1998). Despite the introduction of guidelines to make rTMS safer, these risks remain. Cerebellar rTMS is a potentially safer technique, because it has not been shown to cause seizure activity. This may be because the offset position of the cerebellum means rTMS has a reduced chance of inadvertently stimulating other brain regions. However, as a newer and less studied rTMS variant, cerebellar rTMS has less data on both usage and potential complications. It can be assumed that using the minimum amount of stimulation required to achieve an effect is the safer and preferable approach to any other. Therefore, despite the fact that bilateral cerebellar rTMS has been shown to be more effective than unilateral, it may be in a clinical context the bilateral approach is reserved for patients who do not respond to unilateral rTMS or in whom their dysphagia is particularly severe.

### Limitations

Despite the fact that the primary purpose of this study was to compare the neuronal excitatory and reversal effects of bilateral cerebellar rTMS to unilateral cerebellar rTMS, the lack of a sham arm could be considered a limitation. In particular, the presence of a sham arm would have enabled visualisation of the suppressive motor cortical and behavioural effects of a cortical virtual lesion. However, it is not anticipated that this would have affected the key study findings.

## Conclusion

In conclusion, our studies provide novel pre-clinical physiological data showing that bilateral cerebellar rTMS is more effective at exciting human cortical swallowing pathways than unilateral stimulation alone. This would support the application of bilateral cerebellar rTMS as a viable method of rTMS administration for future clinical trials of dysphagia. More work, however, is still required to explore the neurophysiology of intrinsic cerebellar excitation in the human swallowing system for translation into clinical care. In addition, the effect of combining cerebellar neurostimulation and swallowing skill training for neurorehabilitation should be explored in future studies.
